# Supplementary material for: FBP2—A New Player in Regulation of Motility of Mitochondria and Stability of Microtubules in Cardiomyocytes
Source: Cells. 2022 May 21;11(10):1710. doi: 10.3390/cells11101710 (PMC9139521; doi:10.3390/cells11101710)
Supplement: Supplementary file 1 [file cells-11-01710-s001.zip › cells-1715074-supplementary.pdf]

Supplementary Materials

Table S1. Mass spectrometry identified tubulin chains and MAP1B as potential FBP2 interactors.

Tubulin alpha-1C chain Q3TIZ0|Q3TIZ0_MOUSE
Mass: 50592 Score: 257 Queries matched: 6 emPAI: 0.43
Putative uncharacterized protein OS=Mus musculus GN=**Tuba1c** PE=2 SV=1

Query	Observed	Mr(expt)	Mr(calc)	ppm	Miss	Score	Expect	Rank	Peptide
899	508.2930	1014.5714	1014.5709	0.44	0	61	0.00024	1	K.DVNAAIATIK.T
929	512.2275	1022.4405	1022.4417	-1.14	0	47	0.002	1	K.EDAANNYAR.G
2755	799.8901	1597.7656	1597.7599	3.54	0	70	2.7e-05	1	R.TIQFVDWCPTGFK.V
2941	851.4570	1700.8995	1700.8985	0.60	0	70	2.8e-05	1	R.AVFVDLEPTVIDEVR.T
3175	912.9942	1823.9739	1823.9782	-2.36	0	57	0.00044	1	K.VGINYQPPTVVPGGDLAK.V
3384	1004.4486	2006.8826	2006.8858	-1.57	0	108	1.7e-09	1	K.TIGGGDDSFNTFFSETGAGK.H

Microtubule-associated protein 1B B2RQQ5|B2RQQ5_MOUSE
Mass: 271395 Score: 179 Queries matched: 3 emPAI: 0.03
Microtubule-associated protein 1B OS=Mus musculus GN=**Mtap1b** PE=2 SV=1

Query	Observed	Mr(expt)	Mr(calc)	ppm	Miss	Score	Expect	Rank	Peptide
947	514.8028	1027.5910	1027.5913	-0.28	0	81	1.7e-06	1	R.AVLDALLEGK.A
1638	603.3339	1204.6533	1204.6526	0.62	0	55	0.00092	1	K.LEMYVLNPVK.S
3276	964.4337	1926.8529	1926.8595	-3.46	0	107	2.3e-09	1	R.SSYYVVSGNDPAAEEPSR.A

Tubulin beta-2B chain B2RSN3|B2RSN3_MOUSE
Mass: 50377 Score: 126 Queries matched: 2 emPAI: 0.13
MCG1395 OS=Mus musculus GN=**Tubb2b** PE=2 SV=1

Query	Observed	Mr(expt)	Mr(calc)	ppm	Miss	Score	Expect	Rank	Peptide
2788	808.4231	1614.8316	1614.8287	1.82	0	74	1.3e-05	1	R.AILVDLEPGTMDSVR.S
3978	933.4530	2797.3371	2797.3361	0.33	0	88	2.9e-07	1	R.SGPFGQIFRPDNFVFGQSGAGNNWAK.G

Tubulin beta-4A chain B4F7C2|B4F7C2_RAT
Mass: 50010 Score: 112 Queries matched: 2 emPAI: 0.13
RCG45085 OS=Rattus norvegicus GN=**Tubb4** PE=2 SV=1

Query	Observed	Mr(expt)	Mr(calc)	ppm	Miss	Score	Expect	Rank	Peptide
997	520.3003	1038.5861	1038.5862	-0.10	0	58	0.00027	1	R.YLTVAAVFR.G
3978	933.4530	2797.3371	2797.3361	0.33	0	88	2.9e-07	1	R.SGPFGQIFRPDNFVFGQSGAGNNWAK.G

Tubulin beta-5 chain Q80ZV2|Q80ZV2_MOUSE
Mass: 21554 Score: 69 Queries matched: 2 emPAI: 0.31
Tubb5 protein (Fragment) OS=Mus musculus GN=**Tubb5** PE=2 SV=1

Query	Observed	Mr(expt)	Mr(calc)	ppm	Miss	Score	Expect	Rank	Peptide
997	520.3003	1038.5861	1038.5862	-0.10	0	58	0.00027	1	R.YLTVAAVFR.G
2861	830.4525	1658.8905	1658.8879	1.54	0	43	0.012	1	R.ALTVPELTQQVFDAK.N

Figure S1. Analysis of shape of the mitochondria

The shape of mitochondria was analyzed using the MiNA (Mitochondrial Network Analysis by Sturat Lab) plugin of the ImageJ/FIJI.

The form factor, defined as F = perimeter^2/4π x *area*, is an indicator of the shape of an object. For a perfect circle F=1 and it increases above 1 as the shape of the object becomes more elongated and complex (e.g., more branched). In untreated HL-1 cells (control) as well as in HL-1 cells with partially silenced expression of FBP2 (FBP2-) mitochondrial F values are higher than in HL-1 cells treated with the FBP2-tetramerizing agent (iFBP2), suggesting the FBP2-tetramerization-induced transition from elongated and complex to more simple and round morphology. *** $p < 0.001$.

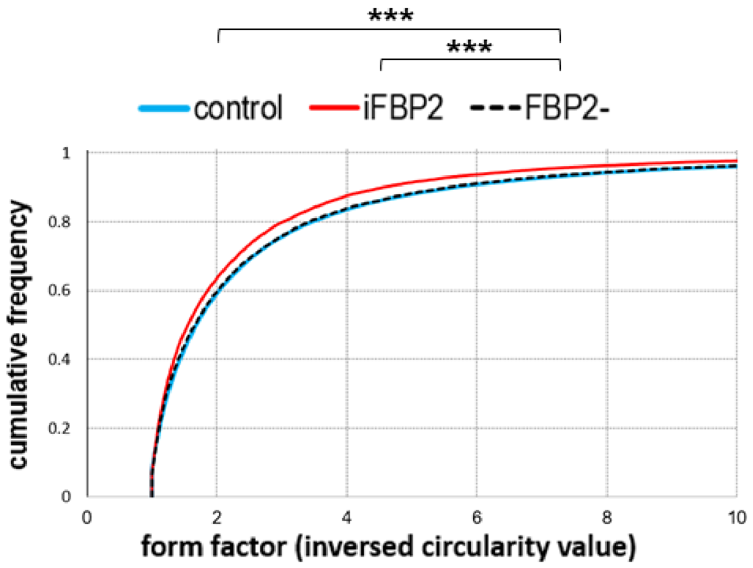

Figure S2. Forced tetramerization of FBP2 does not change sarcomeric organization of actin in HL-1 cells

A) untreated HL-1 cells; B) the FBP2-tetramerizing agent-treated HL-1 cells. Bar = 10 µm.

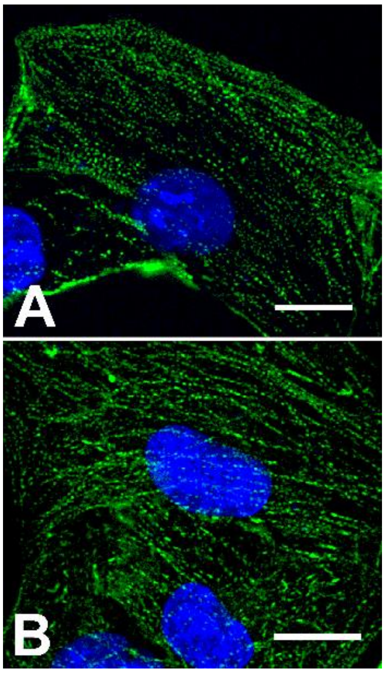

Figure S3. Addition of 5.5 mM glucose to the culture medium does not change the effects of FBP2 tetramerization on mitochondrial motility

Velocity (given in µm/s) of at least 284 mitochondria was measured in each condition. *** p = 0.

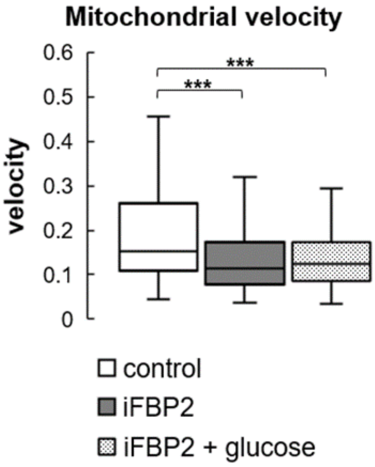

Figure S4. Tetramerization of FBP2 results in a significant shortening of microtubule length in HL-1 cardiomyocytes.

Quantification of the microtubule (MT) length was performed using ImageJ and the modified open source workflow created by Perrine Paul-Gilloteaux and available on the website: Bioimage Informatics Index, https://biii.eu/microtubule-length-analysis.

In each condition, measurements were taken from at least 33 cells from 12 randomly selected areas. *** $p < 0.001$.

Although in the original microscopic image, MTs appear to stretch almost the length of the untreated cell and thus, their length should reach at least dozen micrometers, subsequent steps of the workflow (“tubeness”, “skeletonization”) reveal that MTs create an intertwined network, the sections of which are measured by the program separately. Thus, this workflow probably lowers the MT size under the control conditions, but still shows the clear differences between MTs in cells with the “native” (a mixture of dimeric and tetrameric forms) FBP2 and chemically tetramerized FBP2.

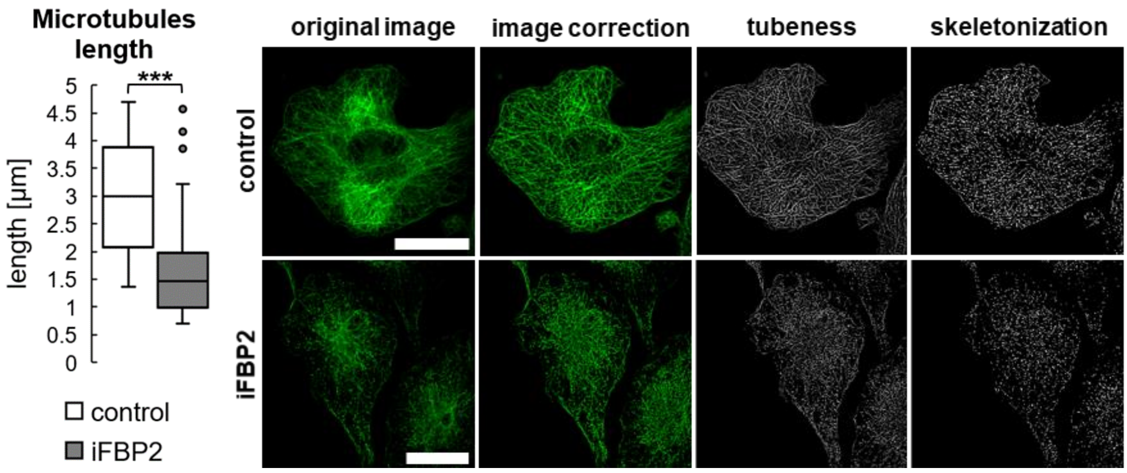

Table S2. Soluble (non-transmembrane) proteins identified with the highest score by mass spectrometry in extracellular vesicles secreted by cardiac fibroblasts into the culture medium

Soluble proteins	Entry number	Score
Heat shock cognate 71 kda protein	P63017	1099
Pyruvate kinase PKM	P52480	769
Heat shock 70kda protein 1B	P17879	614
Heat shock protein hsp 90-beta	P11499	598
Heat shock 70kda protein 1-like	P16627	556
Heat shock protein hsp 90-alpha	P07901	506
Glyceraldehyde-3-phosphate dehydrogenase	P16858	491
Fructose-bisphosphate aldolase A	P05064	437
Protein disulphide-isomerase	P09103	437
**14-3-3 protein zeta/delta**	**P63101**	**333**
Elongation factor 1-alpha 1	P10126	312
L-lactate dehydrogenase b chain	P16215	307
Peptidyl-propyl cis-trans isomerase A	P17742	285
Atp-citrate synthase	Q91V92	281
Phosphoglycerate kinase 1	P09411	270
Udp-gucose 6-dehydrogenase	O70475	268
Peptidyl-prolyl cis-trans isomerase B	P24369	266
Triosephosphate isomerase	P17751	256
Alpha-enolase	P17182	241
Protein disulphide-isomerase A4	P08003	234
L-lactate dehydrogenase a chain	P06151	231
Aldehyde disulphide-isomerase A4	O35945	230
Glucose-6-phosphate 1-dehydrogenase X	Q00612	230
Protein disulphide-isomerase A6	Q922R8	228
Nucleoside diphosphate kinase B	Q01768	210
Elongation factor 2	P58252	197
Protein disulphide-isomerase A3	P27773	197
Retinal dehydrogenase 1	P24549	191
**14-3-3 epsilon protein**	**P62259**	**163**
**14-3-3 gamma protein**	**P61982**	**162**
Neuropilin-1	P97333	155
**14-3-3 theta protein**	**P68254**	**151**
Plastin-3	Q99K51	145
Malate dehydrogenase cytoplasmic	P14152	139
6-phosphogluconate dehydrogenase	Q9DCD0	133
40s ribosomal protein S2	P25444	129
**14-3-3 beta/alpha protein**	**Q9CQV8**	**127**
Serine/arginine-rich splicing factor 1	Q6PDM2	104
